# Reliability and minimal clinically important differences of gait characteristics in peripheral vestibular disorders

**DOI:** 10.3389/fneur.2026.1818995

**Published:** 2026-05-05

**Authors:** Sandra Kollmansperger, Julian Decker, Klaus Jahn, Ken Möhwald, Max Wuehr

**Affiliations:** 1 German Center for Vertigo and Balance Disorders (DSGZ), Ludwig-Maximilians-University of Munich, Munich, Germany; 2 Schön Klinik Bad Aibling, Bad Aibling, Germany; 3 Department of Neurology, Ludwig-Maximilians-University of Munich, Munich, Germany

**Keywords:** gait analysis, gait ataxia, minimal clinically important difference, reliability, vestibulopathy

## Abstract

**Objective:**

Sensory gait ataxia is a core feature of chronic peripheral vestibular disorders, leading to impaired mobility, reduced quality of life, and increased fall risk. Quantitative gait metrics capturing this deficit are valuable endpoints for clinical trials, yet data on their reliability and clinically meaningful change remain limited.

**Methods:**

Gait was recorded in 60 patients with chronic bilateral (*n* = 30) or unilateral (*n* = 30) vestibular failure on an instrumented walkway during self-selected, slow, and eyes-closed walking. Test–retest reliability was quantified using intraclass correlation coefficients across five gait domains: pace, phase, variability, asymmetry, and postural control. Minimal clinically important differences (MCIDs) were estimated using distribution- and anchor-based methods. Anchors included established measures of mobility, fall-related self-efficacy, and quality of life: the Functional Gait Assessment, Falls Efficacy Scale–International, and the Physical Component Score of the Short Form Health Survey.

**Results:**

Self-selected walking showed good-to-excellent reliability across all domains except asymmetry, with similar results for slow and eyes-closed walking. Clinically meaningful changes were linked to faster gait, longer swing phases, lower variability and asymmetry, and narrower stride width, enabling MCID determination for each metric.

**Conclusion:**

Quantitative gait metrics can be reliably assessed in peripheral vestibular disorders and provide robust, clinically relevant endpoints for future intervention studies.

## 1 Introduction

Gait disturbances are a key finding in patients with peripheral vestibular deficits, whether resulting from bilateral dysfunction or insufficient compensation following unilateral loss (1–6). Nearly all affected individuals report chronic unsteadiness and gait insecurity, whereas only a subset of patients [20–50%, depending on the epidemiological study (3, 5, 7, 8)] experience visual symptoms related to impaired vestibulo-ocular gaze stabilization, such as oscillopsia. Gait impairment in this context has been linked to reduced physical and social functioning, limited mobility, and diminished quality of life (9–11). Moreover, it is a major contributor to the elevated fall risk observed in this population (12, 13), with more than half of fall events in prospective studies occurring during walking activities (10).

**Figure 1:**
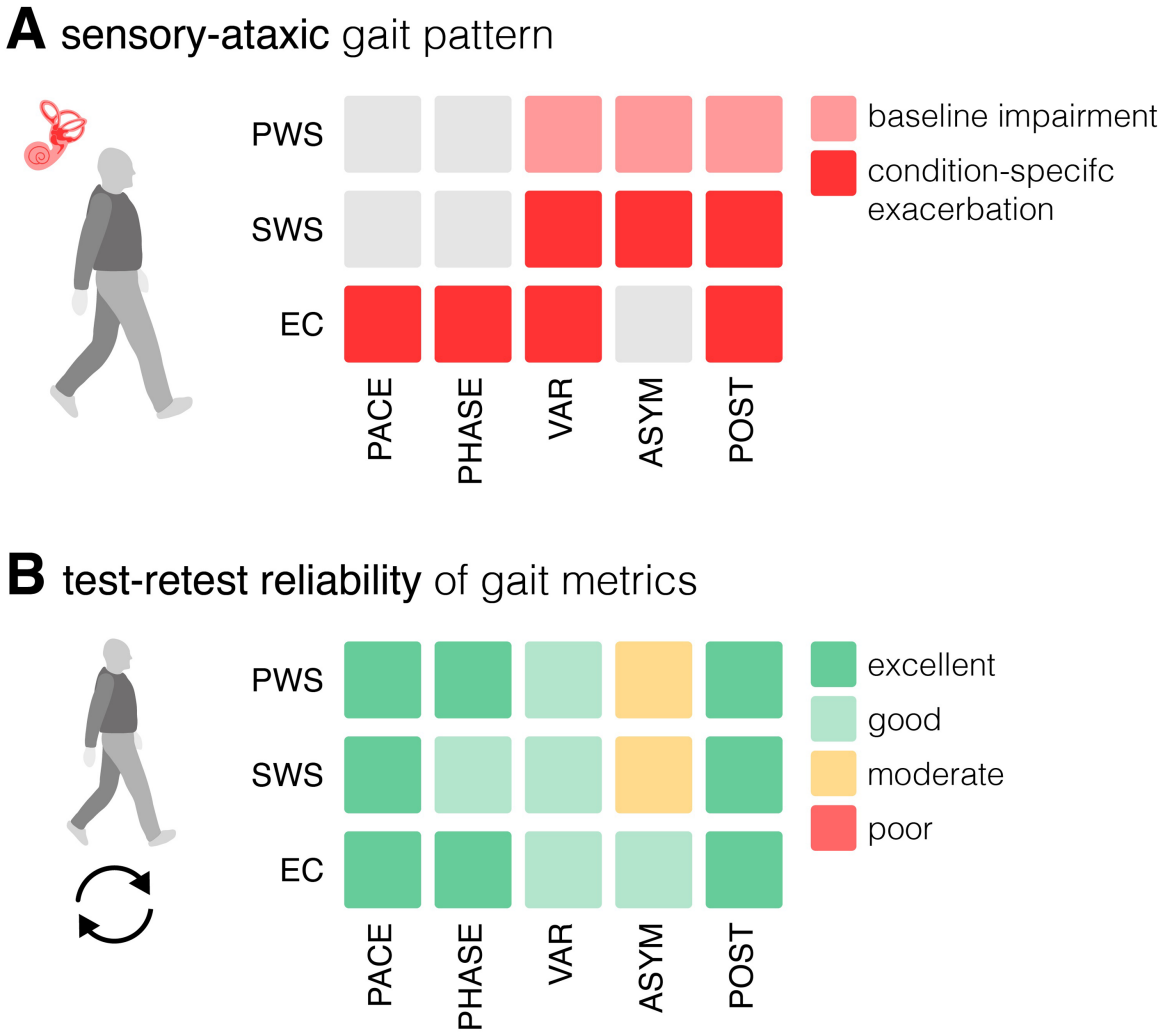
Sensory ataxic gait pattern and reliability of gait metrics. (**A**) Overview matrix illustrating gait alterations associated with sensory ataxia across different walking conditions (PWS, preferred walking speed as baseline, SWS, slow walking speed, EC, eyes closed), grouped by gait domains (pace, phase, variability, asymmetry, postural control), compared to a healthy control group. (**B**) Overview matrix presenting test–retest reliability of gait metrics across different walking conditions and gait domains. Within each domain, the metric with the highest reliability is shown.

The gait disorder in chronic vestibular deficit can generally be characterized as a sensory ataxic gait pattern, with increased variability of step placement due to sensory loss and a compensatory broad-based gait reflecting impaired balance control (Figure 1A) (2, 14, 15). These abnormalities are typically amplified under sensory-challenged conditions, such as walking in the dark or on uneven surfaces. Clinically, these challenges can be simulated by walking with eyes closed. Gait impairments also tend to become more pronounced at slower walking speeds, where greater reliance on vestibular and multisensory feedback is required to maintain dynamic stability (1, 16, 17). Sensory ataxic gait features have emerged as relevant digital mobility markers to characterize disease burden, estimate fall risk, and monitor treatment response in interventions such as vestibular rehabilitation, vestibular implants, or non-invasive vibrotactile or vestibular electric neuromodulation (10, 12, 18–22).

However, despite the clinical relevance of gait assessment in chronic vestibular disorders, systematic data on the test–retest reliability of quantitative gait metrics and their minimal clinically important differences (MCID) remain sparse with few exceptions (23, 24). As a result, gait is often assessed either under overly controlled laboratory conditions (e.g., on a treadmill) (16, 18), or qualitatively using semi-quantitative clinical scores (25–28) – approaches that may fail to capture the characteristic gait abnormalities seen in sensory gait ataxia. This limitation could be overcome by identifying gait parameters that are both robust across repeated measurements and sensitive to clinically meaningful change. In this context, estimates of gait MCIDs could also support future clinical trial design by informing appropriate sample sizes and expected treatment effects (29).

The aim of this study was to systematically evaluate the test–retest reliability of quantitative gait characteristics in patients with chronic unilateral and bilateral vestibular failure. To this end, we repeatedly assessed gait performance under both standard and symptom-enhancing walking conditions (i.e., walking slow or with eyes closed) and analyzed the reliability of a comprehensive set of spatiotemporal gait parameters. Additionally, we estimated MCIDs for each gait metric using established subjective and objective anchor measures of mobility, fall-related self-efficacy, and quality of life, to inform the potential use of gait metrics as robust and clinically meaningful endpoints in future clinical trials targeting chronic vestibular dysfunction.

## 2 Materials and methods

### 2.1 Participants

A total of 60 patients participated in the study (mean age: 61.3 ± 17.3 years; 27 females), including 30 patients with bilateral vestibulopathy (BVP) and 30 with unilateral vestibulopathy (UVP). Detailed demographic and clinical characteristics patients are presented in Table 1. All participants had a clinically confirmed vestibular deficit according to international diagnostic criteria. BVP was defined by a bilaterally pathological video head impulse test (vHIT; gain <0.6) and/or bilaterally reduced or absent caloric responses (sum of maximum slow-phase nystagmus velocities during warm and cold irrigation <0.6°/s) (4). UVP was diagnosed based on a unilaterally reduced vHIT gain (gain <0.7 or a side difference >0.3) or reduced/absent caloric responses on one side (sum of maximum slow-phase nystagmus velocities during warm and cold irrigation <0.6°/s or >25% interaural asymmetry) (30). The UVP had to be present for at least 3 months to ensure a chronic deficit. Additional inclusion criteria included independent walking ability and no clinically evident motor impairment of the lower limbs (e.g., hemiparesis or paraparesis). All patients further underwent a complete neurological and physical examination. Gait performance of patients was compared to that of 30 healthy participants (mean age: 58.1 ± 15.1 years; 15 females).

**Table 1:** Demographics and clinical characteristics.

Demographic and clinical characteristics	Patients with BVP (*N* = 30)	Patients with UVP (*N* = 30)
Age (years)	57.9 ± 19.3	64.8 ± 14.4
Gender (female|male)	15|15	12|18
Etiology	11 idiopathic, 2 Ménière’s disease, 4 antibiotics, 13 other causes	24 vestibular neuritis, 5 idiopathic, 1 Ménière’s disease
*Neuro-otological characteristics*		
vHIT gain	*left*: 0.33 ± 0.22 *right*: 0.32 ± 0.18	*affected*: 0.56 ± 0.24 *unaffected*: 0.88 ± 0.16
vHIT gain difference	0.09 ± 0.13	0.32 ± 0.17
Caloric response (°/s)	*left*: 5.8 ± 6.0 *right*: 5.8 ± 8.7	*affected*: 8.5 ± 8.4 *unaffected*: 34.8 ± 16.1
Caloric response asymmetry (%)	23.7 ± 21.2	66.7 ± 21.0
Peripheral neuropathy (yes|no)	4|26	8|22

BVP, bilateral vestibulopathy; UVP, unilateral vestibulopathy; vHIT, video head impulse test.

### 2.2 Procedures

#### 2.2.1 Gait assessment

Gait assessment was conducted using a 6.7-meter-long pressure-sensitive walkway (GAITRite®, CIR Systems Inc., Sparta, NJ, United States) with a sampling frequency of 120 Hz. Participants were instructed to walk under three different conditions: preferred walking speed (PWS; “Please walk at your normal walking pace”), slow walking speed (SWS; “Please walk as slowly as possible while maintaining a fluid pace”), and walking with eyes closed (EC; “Please walk at your normal walking pace while keeping your eyes closed”). Each condition was recorded four times, and all assessments were performed without the use of ambulatory aids. Step data from two walkway trials were pooled to ensure enough gait cycles for calculating variability and asymmetry markers. The average number of gait cycles per condition was 21.8 ± 4.5 for PWS, 29.2 ± 4.8 for SWS, and 30.4 ± 8.5 for EC, all well above the commonly recommended minimum of 15 cycles for reliable estimation of variability and asymmetry metrics (31). For the two resulting walks per condition, 13 spatiotemporal gait parameters were analyzed, covering five established domains of gait: the pace domain (gait velocity [cm/s], stride length [cm], and stride time [s]); the phase domain (swing and double support phases [% of gait cycle]); the variability domain (coefficient of variation [CV] of stride length [%], stride time [%], and swing phase [%]); the asymmetry domain (left–right asymmetry of stride length [%], stride time [%], and swing phase [%]); and the postural control domain (mean [cm] and CV [%] of base of support).

#### 2.2.2 Assessment of clinical anchors

In addition to instrumented gait analysis, each patient underwent an assessment of functional gait capacity using the Functional Gait Assessment (FGA), an evaluation of subjective balance confidence using the Falls Efficacy Scale-International (FES-I), and a survey of health-related quality of life using the Short Form Health Survey (SF-12). These three measures served as clinical anchors to estimate the magnitude of clinically meaningful change.

The FGA is a 10-item clinical assessment used to evaluate postural stability during several walking tasks, including level-ground walking, head turns while walking, pivot turns, obstacle negotiation, and stair ascent and descent (32). Total scores range from 0 to 30, with lower scores indicating poorer performance. An FGA score below 18 has been associated with considerable gait impairment and an increased risk of falling in older adults and individuals with balance disorders (33). A 4-point increase in FGA score from baseline is considered clinically meaningful in patients with vestibular dysfunction (34).

The FES-I assesses patients’ concern about falling during a broad spectrum of daily activities (35). It includes 16 items addressing both basic and more challenging tasks (e.g., walking on uneven surfaces, reaching overhead, or visiting friends), with total scores ranging from 16 to 64. Higher scores indicate a greater concern about falling. In patients with vestibular disorders, a reduction of 8 points has been shown to represent a clinically meaningful improvement in balance confidence (36).

The SF-12 is a validated instrument for evaluating health-related quality of life across two dimensions: the Physical Component Summary (PCS-12) and the Mental Component Summary (MCS-12), each standardized to a mean of 50 in the general population (37). In patients with chronic vestibulopathy, the PCS-12 has been reported to be reduced, reflecting the impact of physical symptoms such as imbalance and reduced mobility, whereas the MCS-12 typically remains within normal limits (9, 10). Although the reported MCIDs for the PCS-12 vary across studies, a change of more than 10 points has been consistently suggested as an upper threshold for clinically meaningful improvement in patients with neurological movement disorders (38).

### 2.3 Statistical analysis

#### 2.3.1 Preprocessing

Normality of the distributions of gait metrics and clinical anchor variables was assessed using the Shapiro–Wilk test. Outliers exceeding 3 standard deviations from the mean were removed on a pairwise basis to reduce their influence on subsequent analyses; on average, this affected only 1.3% of measurements (range: 0 to 2.8%) across metrics and conditions. All analyses were performed both for the full cohort of 60 patients (reported in the main manuscript) and separately for the BVP and UVP subgroups (reported in the Supplementary material). All statistical analyses were conducted in Python (version 3.13) using the pandas and SciPy libraries.

#### 2.3.2 Comparison to healthy controls

Gait performance of patients was compared to that of healthy controls across all 13 gait metrics and walking conditions (PWS, SWS, EC) using independent-samples *t*-tests.

#### 2.3.3 Reliability analysis

For each walking condition (PWS, SWS, EC), the test–retest reliability of the 13 gait metrics was evaluated across two walks with the intraclass correlation coefficient for absolute agreement (ICC(3,1); two-way mixed-effects model). ICC values were interpreted according to commonly used guidelines: <0.5 = poor, 0.5–0.75 = moderate, 0.75–0.9 = good, and >0.9 = excellent reliability.

#### 2.3.4 Minimal clinically important difference analysis

MCID values were estimated using two established approaches: a distribution-based method and an anchor-based method (39). For the distribution-based analysis, means and standard deviations of each gait metric were computed for each walking condition across the cohort. Effect sizes of 0.2 (small), 0.5 (medium), and 0.8 (large) were multiplied by the standard deviation of each gait parameter to estimate distribution-based MCID thresholds (40, 41).

For the anchor-based MCID estimation, three clinically established anchors were used (see 2.2.2): the FGA, the FES-I, and the PCS-12. These anchors reflect objective mobility/gait performance, fall-related self-efficacy, and health-related quality of life. As recommended in previous literature, only gait metrics and anchors with a minimum linear association (Pearson’s correlation coefficient ∣r∣ > 0.3) were included in the analysis (39). The univariate relationship between each gait metric and anchor variable was estimated using linear regression. Based on this, the MCID of each gait metric was calculated by multiplying the unstandardized regression coefficient (B) by the established MCID of the respective anchor. For example, if the regression coefficient for gait velocity with respect to the FGA is B = 2.0, and the MCID of the FGA is 4 points, the estimated MCID for gait velocity would be 8.0 cm/s. This implies that a clinically meaningful improvement in FGA would correspond to an expected increase of 8.0 cm/s in gait velocity.

In line with previous recommendations, we derived a single, robust, and balanced MCID estimate by triangulating distribution- and anchor-based values across all four metrics: the distribution-based estimate for a medium effect size (0.5 × SD) and the anchor-based MCIDs derived from the FGA, FES-I, and PCS (SF-12) (39, 41, 42). If fewer than four estimates were available for a given metric, the average was calculated from the available values.

#### 2.3.5 Minimal detectable change analysis

In addition, the minimal detectable change at the 95% confidence level (MDC) was calculated to quantify the smallest change exceeding measurement error. The standard error of measurement (SEM) was derived from the ICC and the standard deviation (SD) as $\mathrm{SEM}=\mathrm{SD}\times \sqrt{\left(1-\mathrm{ICC}\right)}$ . MDC was then calculated as $\mathrm{MDC}95=1.96\times \sqrt{2}\times \mathrm{SEM}.$

## 3 Results

### 3.1 Reliability analysis

The results of the reliability analysis are presented in detail in Table 2 and summarized in Figure 1B. Separate, detailed reliability analyses for the subgroups of patients with BVP and UVP are provided in the Supplementary Tables 1, 2, Supplementary Figure 1.

**Table 2:** Reliability analysis outcomes.

Condition	Domain	Metric	Mean ± SD	ICC (3,1)	CI 95%	*F*	*p*-value
Preferred walking speed (PSW)	Pace	vel (cm/s)	98.27 ± 21.27	0.98	[0.95, 0.99]	79.3	<0.001
		slen (cm)	112.52 ± 20.49	0.99	[0.97, 0.99]	144.1	<0.001
		stime (s)	1.16 ± 0.09	0.92	[0.84, 0.96]	23.54	<0.001
	Phase	swing (%)	36.45 ± 1.94	0.95	[0.91, 0.98]	43.08	<0.001
		dsupp (%)	26.96 ± 4.04	0.97	[0.94, 0.99]	68.03	<0.001
	Variability	slen_CV_ (%)	3.49 ± 1.49	0.65	[0.39, 0.81]	4.7	<0.001
		stime_CV_ (%)	3.17 ± 1.28	0.6	[0.32, 0.78]	3.95	<0.001
		swing_CV_ (%)	5.61 ± 2.74	0.83	[0.67, 0.91]	10.45	<0.001
	Asymmetry	slen_ASYM_ (%)	0.71 ± 0.30	0.32	[−0.03, 0.6]	1.94	0.035
		stime_ASYM_ (%)	0.91 ± 0.52	0.51	[0.2, 0.73]	3.08	0.001
		swing_ASYM_ (%)	3.81 ± 2.26	0.55	[0.25, 0.75]	3.43	<0.001
	Postural Control	swidth (cm)	11.48 ± 3.67	0.94	[0.88, 0.97]	32.99	<0.001
		swidth_CV_ (%)	23.80 ± 13.82	0.75	[0.55, 0.87]	7.09	<0.001
Slow walking speed (SWS)	Pace	vel (cm/s)	53.29 ± 10.68	0.86	[0.72, 0.93]	13.2	<0.001
		slen (cm)	83.83 ± 13.09	0.93	[0.86, 0.97]	29.02	<0.001
		stime (s)	1.61 ± 0.23	0.95	[0.89, 0.98]	38.84	<0.001
	Phase	swing (%)	31.79 ± 2.14	0.86	[0.71, 0.93]	12.98	<0.001
		dsupp (%)	36.27 ± 4.15	0.86	[0.73, 0.93]	13.74	<0.001
	Variability	slen_CV_ (%)	5.44 ± 2.08	0.47	[0.13, 0.72]	2.81	0.005
		stime_CV_ (%)	4.94 ± 2.01	0.66	[0.38, 0.83]	4.86	<0.001
		swing_CV_ (%)	11.66 ± 4.92	0.81	[0.64, 0.91]	9.75	<0.001
	Asymmetry	slen_ASYM_ (%)	0.90 ± 0.52	0.65	[0.38, 0.82]	4.76	<0.001
		stime_ASYM_ (%)	0.71 ± 0.43	0.34	[−0.03, 0.63]	2.03	0.036
		swing_ASYM_ (%)	6.65 ± 4.56	0.64	[0.36, 0.82]	4.58	<0.001
	Postural Control	swidth (cm)	13.50 ± 4.32	0.97	[0.94, 0.99]	67.08	<0.001
		swidth_CV_ (%)	18.92 ± 13.29	0.89	[0.78, 0.95]	17.56	<0.001
Walking with eyes closed (EC)	Pace	vel (cm/s)	73.14 ± 18.43	0.88	[0.77, 0.94]	15.81	<0.001
		slen (cm)	84.75 ± 18.73	0.92	[0.84, 0.96]	24.35	<0.001
		stime (s)	1.18 ± 0.13	0.91	[0.82, 0.95]	20.95	<0.001
	Phase	swing (%)	34.27 ± 2.96	0.9	[0.81, 0.95]	19.92	<0.001
		dsupp (%)	31.52 ± 6.15	0.94	[0.88, 0.97]	31.98	<0.001
	Variability	slen_CV_ (%)	9.96 ± 3.72	0.53	[0.22, 0.74]	3.23	<0.001
		stime_CV_ (%)	6.96 ± 2.84	0.76	[0.57, 0.88]	7.48	<0.001
		swing_CV_ (%)	14.53 ± 5.39	0.8	[0.63, 0.9]	9.08	<0.001
	Asymmetry	slen_ASYM_ (%)	1.49 ± 0.81	0.16	[−0.19, 0.48]	1.38	0.185
		stime_ASYM_ (%)	1.26 ± 0.94	0.78	[0.6, 0.89]	8.27	<0.001
		swing_ASYM_ (%)	7.18 ± 5.48	0.66	[0.42, 0.82]	4.97	<0.001
	Postural Control	swidth (cm)	14.94 ± 5.16	0.92	[0.83, 0.96]	22.57	<0.001
		swidth_CV_ (%)	27.04 ± 15.39	0.87	[0.75, 0.94]	14.56	<0.001

SD, standard deviation; ICC, intraclass correlation coefficient; CI, confidence interval; vel, gait velocity; slen, stride length; stime, stride time; swing, swing phase; dsupp, double support phase; swidth, stride width; CV, coefficient of variation; ASYM, asymmetry.

At preferred walking speed, patients exhibited typical features of a sensory ataxic gait pattern, including increased variability (stride length CV, stride time CV, and swing time CV; all *p* < 0.05), greater asymmetry (stride time and swing phase asymmetry; all *p* < 0.05), and a wider base of support (*p* < 0.001) compared to healthy controls. Reliability analysis demonstrated good-to-excellent test–retest reliability not only for gait speed but also for key domains reflecting the severity of gait ataxia, whereas asymmetry metrics showed overall moderate reliability.

At slow walking speed, patients exhibited similarly pronounced features of a sensory ataxic gait pattern, with increased variability (stride length CV and swing phase CV; all *p* < 0.05), greater asymmetry (swing phase asymmetry, *p* = 0.008), and a wider base of support (*p* < 0.001) compared to healthy controls. Test–retest reliability in this condition was largely comparable to preferred walking.

Under eyes-closed walking, patients showed further deterioration of gait performance, characterized by reduced gait velocity and stride length (both *p* < 0.001), prolonged stride time (*p* = 0.025), reduced swing phase (*p* < 0.001), increased double support phase (*p* = 0.003), increased variability (stride length CV, stride time CV, and swing phase CV; all *p* ≤ 0.010), and a wider base of support (*p* = 0.003) compared to healthy controls. This condition also demonstrated good-to-excellent test–retest reliability across all key metric domains, including gait asymmetry.

### 3.2 Minimal detectable change and minimal clinically important difference analysis

A detailed overview of the MDC and MCID estimates is provided in Table 3. Additional subgroup analyses for patients with BVP and UVP are available in Supplementary Tables 3, 4.

**Table 3:** MDC and MCID analysis outcomes.

Condition	Domain	Metric	MDC	MCID						
				Distribution-based			Anchor-based			Triangulated (med. Effect, FGA, FES-I, PCS-12)
				Small effect	Medium effect	Large effect	FGA	FES-I	PCS-12	
Preferred walking speed (PSW)	Pace	vel (cm/s)	5.31	4.25	10.64	17.02	7.18	7.49	11.02	**9.08**
		slen (cm)	3.75	4.10	10.25	16.39	6.17	6.34	9.74	**8.12**
		stime (s)	−0.03	−0.02	−0.04	−0.07	−0.03		−0.04	**−0.04**
	Phase	swing (%)	0.56	0.39	0.97	1.55	0.83	0.65	0.81	**0.82**
		dsupp (%)	−1.05	−0.81	−2.02	−3.23	−1.68	−1.48	−1.82	**−1.75**
	Variability	slen_CV_ (%)	**−1.32**	−0.30	−0.75	−1.19	−0.52	−0.66	−0.59	−0.63
		stime_CV_ (%)	**−0.90**	−0.26	−0.64	−1.02	−0.51	−0.55		−0.57
		swing_CV_ (%)	**−2.03**	−0.55	−1.37	−2.19	−1.25	−1.26	−0.76	−1.16
	Asymmetry	slen_ASYM_ (%)	**−0.54**	−0.06	−0.15	−0.24				−0.15
		stime_ASYM_ (%)	**−0.57**	−0.09	−0.22	−0.35				−0.22
		swing_ASYM_ (%)	**−3.04**	−0.45	−1.12	−1.79	−0.72		−0.90	−0.91
	Postural Control	swidth (cm)	−1.25	−0.74	−1.86	−2.98		−1.79		**−1.83**
		swidth_CV_ (%)	−6.43	−2.81	−7.01	−11.22				**−7.01**
Slow walking speed (SWS)	Pace	vel (cm/s)	5.51	2.14	5.34	8.55			3.27	**4.31**
		slen (cm)	5.15	2.62	6.54	10.47		6.12		**6.33**
		stime (s)	−0.10	−0.04	−0.11	−0.18				**−0.11**
	Phase	swing (%)	**1.48**	0.43	1.07	1.71				1.07
		dsupp (%)	**−2.77**	−0.83	−2.07	−3.32				−2.07
	Variability	slen_CV_ (%)	**−2.28**	−0.42	−1.04	−1.67				−1.04
		stime_CV_ (%)	**−1.79**	−0.40	−1.01	−1.61	−0.54	−0.66	−0.57	−0.69
		swing_CV_ (%)	**−3.73**	−0.98	−2.46	−3.94				−2.46
	Asymmetry	slen_ASYM_ (%)	**−0.68**	−0.10	−0.26	−0.42				−0.26
		stime_ASYM_ (%)	**−0.61**	−0.09	−0.21	−0.34				−0.21
		swing_ASYM_ (%)	**−5.33**	−0.91	−2.28	−3.65			−1.29	−1.78
	Postural Control	swidth (cm)	−0.76	−0.86	−2.16	−3.46		−2.06		**−2.11**
		swidth_CV_ (%)	−4.48	−2.66	−6.65	−10.63				**−6.65**
Walking with eyes closed (EC)	Pace	vel (cm/s)	11.36	3.69	9.22	14.75	6.73	10.48	7.61	**8.51**
		slen (cm)	8.34	3.75	9.37	14.99	5.75	9.61	7.44	**8.04**
		stime (s)	−0.06	−0.03	−0.06	−0.10		−0.04		**−0.05**
	Phase	swing (%)	1.42	0.59	1.48	2.37	0.86	1.66	1.04	**1.26**
		dsupp (%)	−2.48	−1.23	−3.07	−4.92	−1.93	−3.22	−2.06	**−2.57**
	Variability	slen_CV_ (%)	**−4.80**	−0.74	−1.86	−2.97	−1.32			**−1.59**
		stime_CV_ (%)	**−2.03**	−0.57	−1.42	−2.27				**−1.42**
		swing_CV_ (%)	**−4.58**	−1.08	−2.69	−4.31	−1.59	−2.96		**−2.41**
	Asymmetry	slen_ASYM_ (%)	**−1.05**	−0.13	−0.32	−0.51				**−0.32**
		stime_ASYM_ (%)	**−0.65**	−0.17	−0.42	−0.67				**−0.42**
		swing_ASYM_ (%)	**−3.94**	−1.10	−2.75	−4.40		−2.49		**−2.62**
	Postural Control	swidth (cm)	−2.42	−0.96	−2.41	−3.85				**−2.41**
		swidth_CV_ (%)	−8.48	−2.65	−6.62	−10.60				**−6.62**

Distribution-based MCID estimates are reported for small (0.2 × SD), medium (0.5 × SD), and large (0.8 × SD) effect sizes. Anchor-based estimates are included only if the correlation coefficient ∣r∣ > 0.3. Triangulated estimates were calculated as the average of the medium effect size distribution-based MCID and anchor-based estimates from FGA, FES-I, and PCS-12. Triangulated estimates are highlighted in bold; if the MDC exceeds the triangulated estimate, the MDC is instead highlighted as the lower bound for clinically meaningful change. SD, standard deviation; FGA, functional gait assessment; FES-I, Falls Efficacy Scale-International; PCS-12, physical component score of the short form health survey; vel, gait velocity; slen, stride length; stime, stride time; swing, swing phase; dsupp, double support phase; swidth, stride width; CV, coefficient of variation; ASYM, asymmetry; post. Contr., postural control.

Clinically meaningful differences in mobility and gait function – assessed subjectively (FES-I, PCS-12) and objectively (FGA) – were consistently associated with faster gait speed, longer swing phases, reduced variability and asymmetry markers, and narrower stride width. Anchor-based estimates derived from FGA, FES-I, and PCS-12 generally fell within the range of distribution-based estimates assuming a small to medium effect size. When comparing across gait conditions, the estimated MCIDs for slow walking and walking with eyes closed were often substantially higher than those observed at preferred walking speed.

Overall, MCID estimates were generally higher than the corresponding MDC values, indicating that clinically meaningful changes exceeded measurement error. However, notable exceptions were observed particularly for variability and asymmetry metrics, where clinically meaningful changes often approached or fell below the MDC threshold.

## 4 Discussion

In this study, we assessed the test–retest reliability of quantitative gait metrics under normal and sensory-challenged walking conditions in patients with chronic unilateral and bilateral vestibular failure. In addition, we estimated MCIDs for each metric, anchored to established subjective and objective measures of mobility function, fall-related self-efficacy, and quality of life. Our analyses demonstrate good-to-excellent reliability at normal self-selected walking speed across all gait domains except asymmetry – particularly for the key domains of variability and postural control. Importantly, this high reliability extends to sensory-challenged slow and eyes-closed walking. The associated MCID estimates identified for each gait metric under the different walking conditions may help inform the selection of robust and clinically meaningful endpoints for future clinical trials.

It is well-established that the severity of sensory ataxic gait disorder in chronic vestibular failure strongly depends on walking speed (1, 2, 16, 17, 43). Even moderate test–retest variability in gait speed could therefore easily mask clinically meaningful changes in disease-specific gait domains. A key finding of this study is the excellent reproducibility of gait speed in our patient cohort – not only under normal walking conditions but also during sensory-challenged tasks. Whether gait speed itself is systematically altered in chronic vestibular failure remains a matter of debate, with some studies reporting a reduction (2, 10) and others no change or even an increase (1). The identified MCID in our cohort for gait speed during normal walking (~9 cm/s) lies at the lower end of the range reported for neurologic and orthopedic gait disorders (44).

The most characteristic gait pathology in chronic vestibular failure is reflected in the postural control and variability domains, typically presenting as a broad-based gait with increased spatiotemporal gait variability (Figure 1A) (2, 14, 15). Gait variability has emerged as a promising biomarker for fall risk and a reliable measure for capturing the effects of therapeutic interventions in this population (10, 12, 18). Our results show that gait impairments in both posture and variability domains exhibit good-to-excellent test–retest reliability – not only during normal walking but also during slow walking, which amplifies the pathological features. Both walking conditions thus appear well suited to monitor disease-related changes and treatment responses. Previous intervention studies in chronic vestibular failure have shown more pronounced improvements during slow walking, with minimal or no changes at normal pace (18). This would argue in favor of including slow walking tasks in clinical trial assessments. However, MCID estimates for slow walking are in part substantially higher than those for normal walking, which may limit sensitivity to smaller improvements.

Until recently, quantitative gait assessment was largely confined to specialized laboratories and was not routinely integrated into clinical trials for peripheral vestibular disorders – in contrast to semiquantitative clinical gait scores. This is now changing with the broader availability of markerless optical (45–47) and wearable motion analysis systems (48–51), which allow for high-resolution gait characterization both in clinical settings and during patients’ daily life. These technologies strengthen the role of gait metrics as patient-relevant and clinically meaningful endpoints in future studies. The present MCID estimates suggest that clinically important changes in all gait domains relevant to sensory ataxia can be captured with markerless optical motion analysis. While current wearable systems may not yet provide the precision needed to detect subtle changes in postural control (49), they are sufficiently accurate to monitor variability-related changes – which are particularly relevant to fall risk assessment and clinical decision-making.

A limitation of the current analysis is that MCIDs were derived using a cross-sectional (between-person) design, linking gait parameter differences to literature-supported thresholds of clinically meaningful anchor changes. While this approach provides a useful proxy for within-person change, it may underestimate longitudinal sensitivity to change (52). Furthermore, the anchor-based MCID estimates varied considerably across different anchors, and sufficient correlation with the anchors was not observed for every gait metric. As a result, for some metrics, triangulated MCID estimates are based solely on the distribution-based method and should therefore be interpreted with caution. Importantly, for several variability and asymmetry metrics, MCIDs approached or fell below the corresponding MDC values, suggesting limited separability from measurement error at the individual level. In such cases, MDC may approximate clinically relevant change but requires longitudinal validation. This finding likely reflects both the lower test–retest reliability of these metrics and their sensitivity to methodological factors such as walkway length and the resulting number of strides, as shorter walkways yield fewer gait cycles per trial and thereby reduce the stability of variability and asymmetry estimates (31). Consequently, aggregation across multiple trials is required to ensure reliable estimation.

## Data Availability

The raw data supporting the conclusions of this article will be made available by the authors, without undue reservation.
