# Supplementary material for: Reliability and minimal clinically important differences of gait characteristics in peripheral vestibular disorders
Source: Front Neurol. 2026 May 5;17:1818995. doi: 10.3389/fneur.2026.1818995 (PMC13183571; doi:10.3389/fneur.2026.1818995)
Supplement: Supplementary file 1 [file Supplementary_file_1.docx]

**Supplementary Table & Figures**

**
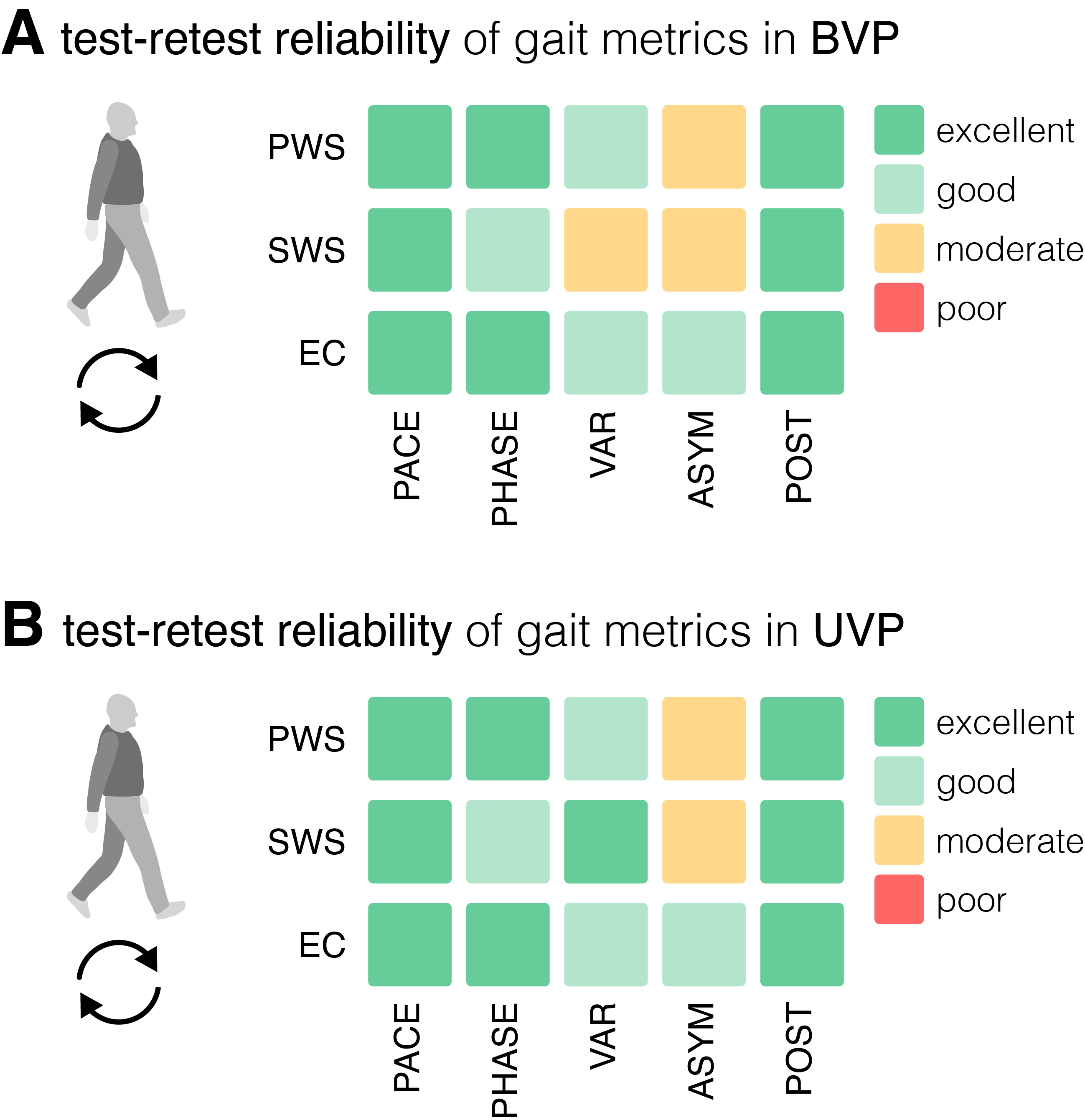
**

**suppl. Figure 1: Sensory ataxic gait pattern and reliability of gait metrics**

(**A**) Overview matrix presenting test-retest reliability of gait metrics across different walking conditions (PWS = preferred walking speed as baseline, SWS = slow walking speed, EC = eyes closed) for patients with bilateral vestibulopathy (BVP), grouped by gait domains (pace, phase, variability, asymmetry, postural control). Within each domain, the metric with the highest reliability is shown. (**B**) Analogous matrix for patients with unilateral vestibulopathy (UVP).

| **condition** | **domain** | **metric** | **mean ± SD** | **ICC (3,1)** | **CI 95%** | **F** | **p** |
| --- | --- | --- | --- | --- | --- | --- | --- |
| **preferred walking speed**  **(PSW)** | **pace** | vel (cm/s) | 101.90 ± 21.38 | 0.98 | [0.96 0.99] | 120.6 | < 0.001 |
|  |  | slen (cm) | 115.84 ± 20.65 | 0.99 | [0.97 1. ] | 198.55 | < 0.001 |
|  |  | stime (s) | 1.15 ± 0.08 | 0.94 | [0.87 0.98] | 35.19 | < 0.001 |
|  | **phase** | swing (%) | 36.76 ± 1.47 | 0.97 | [0.92 0.99] | 61.8 | < 0.001 |
|  |  | dsupp (%) | 26.25 ± 2.98 | 0.98 | [0.94 0.99] | 79.9 | < 0.001 |
|  | **variability** | slen_CV_ (%) | 3.24 ± 1.35 | 0.54 | [0.14 0.79] | 3.36 | 0.006 |
|  |  | stime_CV_ (%) | 2.96 ± 0.99 | 0.67 | [0.34 0.86] | 5.13 | < 0.001 |
|  |  | swing_CV_ (%) | 5.22 ± 2.51 | 0.79 | [0.54 0.91] | 8.53 | < 0.001 |
|  | **asymmetry** | slen_ASYM_ (%) | 0.75 ± 0.34 | 0.34 | [-0.11 0.67] | 2.01 | 0.069 |
|  |  | stime_ASYM_ (%) | 0.90 ± 0.57 | 0.46 | [0.03 0.74] | 2.71 | 0.018 |
|  |  | swing_ASYM_ (%) | 3.83 ± 2.02 | 0.58 | [0.2 0.81] | 3.78 | 0.003 |
|  | **post. contr.** | swidth (cm) | 11.22 ± 3.10 | 0.91 | [0.78 0.96] | 20.95 | < 0.001 |
|  |  | swidth_CV_ (%) | 23.70 ± 10.48 | 0.55 | [0.15 0.79] | 3.45 | 0.005 |
| **slow walking speed**  **(SWS)** | **pace** | vel (cm/s) | 53.15 ± 9.56 | 0.9 | [0.76 0.96] | 19.53 | < 0.001 |
|  |  | slen (cm) | 85.15 ± 13.36 | 0.96 | [0.91 0.99] | 55.7 | < 0.001 |
|  |  | stime (s) | 1.63 ± 0.23 | 0.96 | [0.9 0.99] | 53.12 | < 0.001 |
|  | **phase** | swing (%) | 31.84 ± 1.87 | 0.84 | [0.63 0.94] | 11.79 | < 0.001 |
|  |  | dsupp (%) | 36.11 ± 3.46 | 0.85 | [0.64 0.94] | 12.26 | < 0.001 |
|  | **variability** | slen_CV_ (%) | 5.75 ± 2.13 | 0.37 | [-0.11 0.71] | 2.16 | 0.061 |
|  |  | stime_CV_ (%) | 5.01 ± 2.27 | 0.64 | [0.27 0.85] | 4.63 | 0.001 |
|  |  | swing_CV_ (%) | 11.93 ± 4.92 | 0.74 | [0.44 0.9 ] | 6.83 | < 0.001 |
|  | **asymmetry** | slen_ASYM_ (%) | 1.03 ± 0.52 | 0.66 | [0.29 0.86] | 4.84 | 0.001 |
|  |  | stime_ASYM_ (%) | 0.77 ± 0.43 | 0.19 | [-0.29 0.59] | 1.47 | 0.217 |
|  |  | swing_ASYM_ (%) | 6.26 ± 4.54 | 0.68 | [0.33 0.87] | 5.34 | < 0.001 |
|  | **post. contr.** | swidth (cm) | 13.79 ± 4.53 | 0.98 | [0.95 0.99] | 103.59 | < 0.001 |
|  |  | swidth_CV_ (%) | 19.32 ± 12.93 | 0.86 | [0.67 0.95] | 13.47 | < 0.001 |
| **walking with eyes closed (EC)** | **pace** | vel (cm/s) | 73.00 ± 17.99 | 0.88 | [0.72 0.95] | 15.63 | < 0.001 |
|  |  | slen (cm) | 83.61 ± 18.42 | 0.92 | [0.81 0.97] | 23.7 | < 0.001 |
|  |  | stime (s) | 1.16 ± 0.13 | 0.92 | [0.82 0.97] | 24.79 | < 0.001 |
|  | **phase** | swing (%) | 34.10 ± 2.95 | 0.9 | [0.77 0.96] | 19.55 | < 0.001 |
|  |  | dsupp (%) | 31.92 ± 6.00 | 0.94 | [0.86 0.98] | 32.89 | < 0.001 |
|  | **variability** | slen_CV_ (%) | 9.60 ± 3.66 | 0.66 | [0.32 0.85] | 4.88 | < 0.001 |
|  |  | stime_CV_ (%) | 7.49 ± 2.92 | 0.74 | [0.46 0.89] | 6.77 | < 0.001 |
|  |  | swing_CV_ (%) | 15.70 ± 5.60 | 0.81 | [0.58 0.92] | 9.51 | < 0.001 |
|  | **asymmetry** | slen_ASYM_ (%) | 1.47 ± 0.67 | 0.04 | [-0.4 0.47] | 1.09 | 0.428 |
|  |  | stime_ASYM_ (%) | 1.31 ± 0.85 | 0.76 | [0.5 0.9] | 7.48 | < 0.001 |
|  |  | swing_ASYM_ (%) | 7.86 ± 5.45 | 0.67 | [0.33 0.85] | 5.04 | < 0.001 |
|  | **post. contr.** | swidth (cm) | 16.29 ± 4.65 | 0.9 | [0.77 0.96] | 19.36 | < 0.001 |
|  |  | swidth_CV_ (%) | 24.92 ± 10.17 | 0.82 | [0.61 0.93] | 10.31 | < 0.001 |

**suppl. Table 1: Reliability analysis outcomes for patients with bilateral vestibulopathy**

*Abbreviations: SD – standard deviation; ICC – intraclass correlation coefficient; CI – confidence interval; vel – gait velocity; slen – stride length; stime – stride time; swing – swing phase; dsupp – double support phase; swidth – stride width; CV – coefficient of variation; ASYM – asymmetry; post. contr. – postural control*

| **condition** | **domain** | **metric** | **mean ± SD** | **ICC (3,1)** | **CI 95%** | **F** | **p** |
| --- | --- | --- | --- | --- | --- | --- | --- |
| **preferred walking speed**  **(PSW)** | **pace** | vel (cm/s) | 92.81 ± 20.66 | 0.95 | [0.85 0.99] | 42.61 | < 0.001 |
|  |  | slen (cm) | 107.54 ± 19.94 | 0.98 | [0.92 0.99] | 79.71 | < 0.001 |
|  |  | stime (s) | 1.18 ± 0.09 | 0.87 | [0.6 0.96] | 13.93 | < 0.001 |
|  | **phase** | swing (%) | 35.99 ± 2.48 | 0.95 | [0.84 0.99] | 41.18 | < 0.001 |
|  |  | dsupp (%) | 28.02 ± 5.20 | 0.97 | [0.9 0.99] | 66.88 | < 0.001 |
|  | **variability** | slen_CV_ (%) | 3.86 ± 1.66 | 0.75 | [0.34 0.92] | 7.09 | 0.001 |
|  |  | stime_CV_ (%) | 3.48 ± 1.61 | 0.52 | [-0.04 0.84] | 3.21 | 0.033 |
|  |  | swing_CV_ (%) | 6.20 ± 3.06 | 0.87 | [0.6 0.96] | 13.82 | < 0.001 |
|  | **asymmetry** | slen_ASYM_ (%) | 0.65 ± 0.22 | 0.15 | [-0.44 0.65] | 1.36 | 0.308 |
|  |  | stime_ASYM_ (%) | 0.93 ± 0.44 | 0.65 | [0.16 0.89] | 4.79 | 0.008 |
|  |  | swing_ASYM_ (%) | 3.79 ± 2.65 | 0.55 | [-0.01 0.84] | 3.4 | 0.027 |
|  | **post. contr.** | swidth (cm) | 11.88 ± 4.49 | 0.97 | [0.91 0.99] | 76.03 | < 0.001 |
|  |  | swidth_CV_ (%) | 23.96 ± 18.19 | 0.97 | [0.91 0.99] | 73.24 | < 0.001 |
| **slow walking speed**  **(SWS)** | **pace** | vel (cm/s) | 53.52 ± 12.85 | 0.82 | [0.44 0.95] | 10.4 | < 0.001 |
|  |  | slen (cm) | 81.48 ± 12.76 | 0.86 | [0.55 0.96] | 13.72 | < 0.001 |
|  |  | stime (s) | 1.57 ± 0.22 | 0.92 | [0.73 0.98] | 25.6 | < 0.001 |
|  | **phase** | swing (%) | 31.71 ± 2.64 | 0.88 | [0.59 0.97] | 15.44 | < 0.001 |
|  |  | dsupp (%) | 36.55 ± 5.30 | 0.88 | [0.6 0.97] | 16.05 | < 0.001 |
|  | **variability** | slen_CV_ (%) | 4.89 ± 1.97 | 0.65 | [0.07 0.9 ] | 4.66 | 0.016 |
|  |  | stime_CV_ (%) | 4.81 ± 1.53 | 0.73 | [0.24 0.93] | 6.54 | 0.005 |
|  |  | swing_CV_ (%) | 11.18 ± 5.09 | 0.91 | [0.7 0.98] | 22.5 | < 0.001 |
|  | **asymmetry** | slen_ASYM_ (%) | 0.68 ± 0.47 | 0.59 | [-0.02 0.88] | 3.83 | 0.029 |
|  |  | stime_ASYM_ (%) | 0.60 ± 0.42 | 0.63 | [0.05 0.89] | 4.44 | 0.018 |
|  |  | swing_ASYM_ (%) | 7.33 ± 4.69 | 0.55 | [-0.08 0.86] | 3.41 | 0.041 |
|  | **post. contr.** | swidth (cm) | 12.99 ± 4.05 | 0.91 | [0.67 0.98] | 20.07 | < 0.001 |
|  |  | swidth_CV_ (%) | 18.20 ± 14.42 | 0.97 | [0.9 0.99] | 77.99 | < 0.001 |
| **walking with eyes closed (EC)** | **pace** | vel (cm/s) | 73.35 ± 19.76 | 0.9 | [0.68 0.97] | 18.57 | < 0.001 |
|  |  | slen (cm) | 86.46 ± 19.76 | 0.93 | [0.77 0.98] | 26.21 | < 0.001 |
|  |  | stime (s) | 1.20 ± 0.13 | 0.89 | [0.66 0.97] | 16.84 | < 0.001 |
|  | **phase** | swing (%) | 34.52 ± 3.08 | 0.9 | [0.7 0.97] | 19.93 | < 0.001 |
|  |  | dsupp (%) | 30.92 ± 6.53 | 0.94 | [0.79 0.98] | 30.19 | < 0.001 |
|  | **variability** | slen_CV_ (%) | 10.49 ± 3.87 | 0.42 | [-0.17 0.79] | 2.44 | 0.077 |
|  |  | stime_CV_ (%) | 6.16 ± 2.61 | 0.79 | [0.42 0.93] | 8.56 | < 0.001 |
|  |  | swing_CV_ (%) | 12.78 ± 4.69 | 0.72 | [0.28 0.91] | 6.18 | 0.003 |
|  | **asymmetry** | slen_ASYM_ (%) | 1.53 ± 1.00 | 0.44 | [-0.15 0.8 ] | 2.54 | 0.068 |
|  |  | stime_ASYM_ (%) | 1.18 ± 1.09 | 0.79 | [0.43 0.94] | 8.66 | < 0.001 |
|  |  | swing_ASYM_ (%) | 6.15 ± 5.57 | 0.67 | [0.18 0.89] | 4.99 | 0.006 |
|  | **post. contr.** | swidth (cm) | 12.93 ± 5.39 | 0.91 | [0.71 0.97] | 20.27 | < 0.001 |
|  |  | swidth_CV_ (%) | 30.22 ± 21.02 | 0.9 | [0.69 0.97] | 19.04 | < 0.001 |

**suppl. Table 2: Reliability analysis outcomes for patients with unilateral vestibulopathy**

*Abbreviations: SD – standard deviation; ICC – intraclass correlation coefficient; CI – confidence interval; vel – gait velocity; slen – stride length; stime – stride time; swing – swing phase; dsupp – double support phase; swidth – stride width; CV – coefficient of variation; ASYM – asymmetry; post. contr. – postural control*

| **condition** | **domain** | **metric** | **MDC** | | **MCID** | | | | | |  | | | | | |  | |  |
| --- | --- | --- | --- | --- | --- | --- | --- | --- | --- | --- | --- | --- | --- | --- | --- | --- | --- | --- | --- |
|  |  |  |  | | **distribution-based** | | | | | | **anchor-based** | | | | | | **triangulated**  (med. effect, FGA, FES-I, PCS-12) | |  |
|  |  |  |  | | small effect | | medium effect | | large effect | | FGA | | FES-I | | PCS-12 | |  |  |  |
| **preferred walking speed**  **(PSW)** | **pace** | vel (cm/s) | | 5.44 | | 4.28 | | 10.69 | | 17.10 | |  | |  | | 12.76 | | **11.73** | |
|  |  | slen (cm) | | 3.45 | | 4.13 | | 10.33 | | 16.52 | |  | |  | | 11.50 | | **10.91** | |
|  |  | stime (s) | | -0.03 | | -0.02 | | -0.04 | | -0.07 | | -0.02 | |  | | -0.03 | | **-0.03** | |
|  | **phase** | swing (%) | | 0.53 | | 0.29 | | 0.73 | | 1.18 | |  | |  | | 0.66 | | **0.70** | |
|  |  | dsupp (%) | | -0.94 | | -0.60 | | -1.49 | | -2.38 | |  | | -0.96 | | -1.58 | | **-1.34** | |
|  | **variability** | slen_CV_ (%) | | **-1.45** | | -0.27 | | -0.68 | | -1.08 | |  | |  | | -0.68 | | -0.68 | |
|  |  | stime_CV_ (%) | | **-0.93** | | -0.20 | | -0.50 | | -0.79 | |  | | -0.34 | | -0.28 | | -0.37 | |
|  |  | swing_CV_ (%) | | **-1.79** | | -0.50 | | -1.26 | | -2.01 | |  | | -0.81 | | -0.86 | | -0.98 | |
|  | **asymmetry** | slen_ASYM_ (%) | | **-0.56** | | -0.07 | | -0.17 | | -0.27 | | -0.14 | |  | |  | | -0.15 | |
|  |  | stime_ASYM_ (%) | | **-0.71** | | -0.11 | | -0.29 | | -0.46 | |  | |  | |  | | -0.29 | |
|  |  | swing_ASYM_ (%) | | **-2.65** | | -0.41 | | -1.01 | | -1.62 | | -0.60 | |  | | -0.74 | | -0.78 | |
|  | **post. contr.** | swidth (cm) | | -1.30 | | -0.62 | | -1.55 | | -2.48 | |  | | -1.48 | |  | | **-1.51** | |
|  |  | swidth_CV_ (%) | | **-12.22** | | -2.10 | | -5.24 | | -8.38 | |  | |  | |  | | -5.24 | |
| **slow walking speed**  **(SWS)** | **pace** | vel (cm/s) | | **5.01** | | 1.91 | | 4.78 | | 7.65 | |  | |  | | 3.22 | | 4.00 | |
|  |  | slen (cm) | | 5.04 | | 2.67 | | 6.68 | | 10.69 | |  | | 5.68 | |  | | **6.18** | |
|  |  | stime (s) | | -0.09 | | -0.05 | | -0.12 | | -0.18 | |  | |  | |  | | **-0.12** | |
|  | **phase** | swing (%) | | **1.60** | | 0.37 | | 0.93 | | 1.49 | |  | |  | |  | | **0.93** | |
|  |  | dsupp (%) | | **-2.56** | | -0.69 | | -1.73 | | -2.77 | |  | |  | |  | | **-1.73** | |
|  | **variability** | slen_CV_ (%) | | **-2.95** | | -0.42 | | -1.06 | | -1.70 | |  | |  | |  | | -1.06 | |
|  |  | stime_CV_ (%) | | **-2.12** | | -0.45 | | -1.14 | | -1.82 | | -0.66 | | -0.78 | | -0.82 | | -0.85 | |
|  |  | swing_CV_ (%) | | **-4.04** | | -0.98 | | -2.46 | | -3.93 | |  | |  | | -1.80 | | -2.13 | |
|  | **asymmetry** | slen_ASYM_ (%) | | **-0.69** | | -0.10 | | -0.26 | | -0.41 | |  | |  | |  | | -0.26 | |
|  |  | stime_ASYM_ (%) | | **-0.63** | | -0.09 | | -0.21 | | -0.34 | |  | |  | |  | | -0.21 | |
|  |  | swing_ASYM_ (%) | | **-5.71** | | -0.70 | | -1.75 | | -2.80 | |  | |  | | -1.17 | | -1.46 | |
|  | **post. contr.** | swidth (cm) | | -0.87 | | -0.85 | | -2.12 | | -3.39 | |  | | -2.35 | |  | | **-2.24** | |
|  |  | swidth_CV_ (%) | | **6.70** | | -2.62 | | -6.56 | | -10.50 | |  | |  | |  | | -6.56 | |
| **walking with eyes closed (EC)** | **pace** | vel (cm/s) | | **9.00** | | 3.60 | | 9.00 | | 14.40 | |  | | 9.27 | | 5.85 | | 8.04 | |
|  |  | slen (cm) | | 6.07 | | 3.69 | | 9.21 | | 14.74 | |  | | 8.88 | | 6.17 | | **8.09** | |
|  |  | stime (s) | | **-0.07** | | -0.03 | | -0.06 | | -0.10 | | -0.04 | | -0.04 | |  | | -0.05 | |
|  | **phase** | swing (%) | | 1.18 | | 0.59 | | 1.47 | | 2.36 | |  | | 1.61 | |  | | **1.54** | |
|  |  | dsupp (%) | | -2.12 | | -1.20 | | -3.00 | | -4.80 | |  | | -3.08 | |  | | **-3.04** | |
|  | **variability** | slen_CV_ (%) | | **-3.73** | | -0.73 | | -1.83 | | -2.93 | |  | |  | |  | | -1.83 | |
|  |  | stime_CV_ (%) | | **-2.28** | | -0.58 | | -1.46 | | -2.33 | | -0.90 | | -1.16 | |  | | -1.17 | |
|  |  | swing_CV_ (%) | | **-4.14** | | -1.12 | | -2.80 | | -4.48 | |  | | -3.27 | |  | | -3.04 | |
|  | **asymmetry** | slen_ASYM_ (%) | | **-1.01** | | -0.14 | | -0.34 | | -0.54 | | -0.22 | |  | |  | | -0.28 | |
|  |  | stime_ASYM_ (%) | | **-0.57** | | -0.17 | | -0.42 | | -0.68 | | -0.26 | |  | |  | | -0.34 | |
|  |  | swing_ASYM_ (%) | | **-4.38** | | -1.09 | | -2.73 | | -4.36 | |  | | -3.01 | |  | | -2.87 | |
|  | **post. contr.** | swidth (cm) | | **-2.63** | | -0.93 | | -2.33 | | -3.72 | |  | | -2.22 | |  | | -2.27 | |
|  |  | swidth_CV_ (%) | **-8.96** | | | -2.03 | -5.08 | | -8.13 | |  | | -4.93 | |  | | -5.01 | |  |

**suppl. Table 3: MDC and MCID analysis outcomes for patients with bilateral vestibulopathy**

Distribution-based MCID estimates are reported for small (0.2 × SD), medium (0.5 × SD), and large (0.8 × SD) effect sizes. Anchor-based estimates are included only if the correlation coefficient $|r|$ > 0.3. Triangulated estimates were calculated as the average of the medium effect size distribution-based MCID and anchor-based estimates from FGA, FES-I, and PCS-12. Triangulated estimates are highlighted in bold; if the MDC exceeds the triangulated estimate, the MDC is instead highlighted as the lower bound for clinically meaningful change. *Abbreviations: SD – standard deviation; FGA – functional gait assessment; FES-I – Falls Efficacy Scale-International; PCS-12 – physical component score of the short form health survey; vel – gait velocity; slen – stride length; stime – stride time; swing – swing phase; dsupp – double support phase; swidth – stride width; CV – coefficient of variation; ASYM – asymmetry; post. contr. – postural control*

| **condition** | **domain** | **metric** | **MDC** | **MCID** | | |  | | |  |
| --- | --- | --- | --- | --- | --- | --- | --- | --- | --- | --- |
|  |  |  |  | **distribution-based** | | | **anchor-based** | | | **triangulated**  (med. effect, FGA, FES-I, PCS-12) |
|  |  |  |  | small effect | medium effect | large effect | FGA | FES-I | PCS-12 |  |
| **preferred walking speed**  **(PSW)** | **pace** | vel (cm/s) | 3.78 | 4.13 | 10.33 | 16.53 | 11.44 | 11.06 | 12.49 | **11.33** |
|  |  | slen (cm) | 2.39 | 3.99 | 9.97 | 15.96 | 11.44 | 9.65 | 10.56 | **10.41** |
|  |  | stime (s) | -0.02 | -0.02 | -0.04 | -0.07 | -0.03 |  | -0.06 | **-0.04** |
|  | **phase** | swing (%) | 0.54 | 0.50 | 1.24 | 1.99 | 1.50 | 1.13 | 1.49 | **1.34** |
|  |  | dsupp (%) | -0.80 | -1.04 | -2.60 | -4.16 | -3.17 | -2.40 | -3.11 | **-2.82** |
|  | **variability** | slen_CV_ (%) | -0.93 | -0.33 | -0.83 | -1.33 | -1.11 | -1.50 | -0.76 | **-1.05** |
|  |  | stime_CV_ (%) | **-0.88** | -0.32 | -0.80 | -1.28 | -0.88 | -0.90 | -0.64 | -0.81 |
|  |  | swing_CV_ (%) | 0.24 | -0.61 | -1.53 | -2.45 | -2.27 | -2.69 | -1.05 | **-1.88** |
|  | **asymmetry** | slen_ASYM_ (%) | **-0.36** | -0.04 | -0.11 | -0.17 | -0.06 | -0.16 | -0.09 | -0.10 |
|  |  | stime_ASYM_ (%) | **-0.36** | -0.09 | -0.22 | -0.35 |  | -0.20 |  | -0.21 |
|  |  | swing_ASYM_ (%) | **-1.55** | -0.53 | -1.32 | -2.12 | -0.90 | -1.08 | -1.24 | -1.14 |
|  | **post. contr.** | swidth (cm) | -0.58 | -0.90 | -2.25 | -3.60 | -1.69 | -2.42 |  | **-2.12** |
|  |  | swidth_CV_ (%) | -3.34 | -3.64 | -9.09 | -14.55 |  |  |  | **-9.09** |
| **slow walking speed**  **(SWS)** | **pace** | vel (cm/s) | 5.42 | 2.57 | 6.42 | 10.28 |  |  |  | **6.42** |
|  |  | slen (cm) | 3.54 | 2.55 | 6.38 | 10.21 | 5.18 | 6.01 | 4.48 | **5.51** |
|  |  | stime (s) | -0.08 | -0.04 | -0.11 | -0.18 |  |  |  | **-0.11** |
|  | **phase** | swing (%) | 0.43 | 0.53 | 1.32 | 2.11 | 0.83 |  |  | **1.08** |
|  |  | dsupp (%) | -0.96 | -1.06 | -2.65 | -4.24 | -1.73 |  |  | **-2.19** |
|  | **variability** | slen_CV_ (%) | **-1.30** | -0.39 | -0.98 | -1.57 | -0.65 |  | -0.74 | -0.79 |
|  |  | stime_CV_ (%) | **-0.95** | -0.31 | -0.76 | -1.22 | -0.42 |  |  | -0.59 |
|  |  | swing_CV_ (%) | 2.16 | -1.02 | -2.54 | -4.07 | -1.84 | -2.49 |  | **-2.29** |
|  | **asymmetry** | slen_ASYM_ (%) | **-0.26** | -0.09 | -0.24 | -0.38 |  | -0.21 | -0.17 | -0.20 |
|  |  | stime_ASYM_ (%) | **-0.25** | -0.08 | -0.21 | -0.33 |  |  | -0.26 | -0.23 |
|  |  | swing_ASYM_ (%) | **-3.53** | -0.94 | -2.35 | -3.75 | -2.38 |  | -2.17 | -2.30 |
|  | **post. contr.** | swidth (cm) | 0.52 | -0.81 | -2.02 | -3.24 | -1.76 | -1.82 |  | **-1.87** |
|  |  | swidth_CV_ (%) | 1.91 | -2.88 | -7.21 | -11.54 |  | -4.56 |  | **-5.88** |
| **walking with eyes closed (EC)** | **pace** | vel (cm/s) | 5.88 | 3.95 | 9.88 | 15.81 | 11.28 | 18.84 | 11.83 | **12.96** |
|  |  | slen (cm) | 6.84 | 3.95 | 9.88 | 15.81 | 12.24 | 15.99 | 9.68 | **11.95** |
|  |  | stime (s) | -0.03 | -0.03 | -0.06 | -0.10 |  | -0.06 | -0.07 | **-0.06** |
|  | **phase** | swing (%) | 0.93 | 0.62 | 1.54 | 2.47 | 2.01 | 2.42 | 1.70 | **1.92** |
|  |  | dsupp (%) | -1.48 | -1.31 | -3.27 | -5.23 | -4.23 | -5.03 | -3.49 | **-4.00** |
|  | **variability** | slen_CV_ (%) | **-3.26** | -0.77 | -1.93 | -3.10 | -2.01 | -2.11 | -2.05 | -2.03 |
|  |  | stime_CV_ (%) | **-1.28** | -0.52 | -1.31 | -2.09 | -0.76 |  | -1.18 | -1.08 |
|  |  | swing_CV_ (%) | 2.37 | -0.94 | -2.35 | -3.75 | -2.53 | -4.59 |  | **-3.16** |
|  | **asymmetry** | slen_ASYM_ (%) | **-0.71** | -0.20 | -0.50 | -0.80 |  |  | -0.35 | -0.42 |
|  |  | stime_ASYM_ (%) | -0.40 | -0.22 | -0.54 | -0.87 |  |  |  | **-0.54** |
|  |  | swing_ASYM_ (%) | -1.24 | -1.11 | -2.79 | -4.46 | -2.36 | -2.60 | -1.63 | -1.53 |
|  | **post. contr.** | swidth (cm) | 0.82 | -1.08 | -2.69 | -4.31 | -1.89 |  |  | **-2.29** |
|  |  | swidth_CV_ (%) | 3.40 | -4.20 | -10.51 | -16.82 |  |  |  | **-10.51** |

**suppl. Table 4: MDC and MCID analysis outcomes for patients with unilateral vestibulopathy**

Distribution-based MCID estimates are reported for small (0.2 × SD), medium (0.5 × SD), and large (0.8 × SD) effect sizes. Anchor-based estimates are included only if the correlation coefficient $|r|$ > 0.3. Triangulated estimates were calculated as the average of the medium effect size distribution-based MCID and anchor-based estimates from FGA, FES-I, and PCS-12. Triangulated estimates are highlighted in bold; if the MDC exceeds the triangulated estimate, the MDC is instead highlighted as the lower bound for clinically meaningful change. *Abbreviations: SD – standard deviation; FGA – functional gait assessment; FES-I – Falls Efficacy Scale-International; PCS-12 – physical component score of the short form health survey; vel – gait velocity; slen – stride length; stime – stride time; swing – swing phase; dsupp – double support phase; swidth – stride width; CV – coefficient of variation; ASYM – asymmetry; post. contr. – postural control*
